# Undisclosed Antiretroviral Therapy Use at Primary Health Care Clinics in Rural KwaZulu Natal South Africa: A DO-ART Trial Sub-study

**DOI:** 10.1007/s10461-021-03319-4

**Published:** 2021-06-07

**Authors:** Nsika Sithole, Resign Gunda, Olivier Koole, Meighan Krows, Torin Schaafsma, Mosa Moshabela, Mark J. Siedner, Connie Celum, Ruanne V. Barnabas

**Affiliations:** 1 Africa Health Research Institute, Somkhele campus, R618 en Route To Hlabisa, Mtubatuba, 3935 KwaZulu-Natal South Africa; 2 School of Nursing and Public Health, University of KwaZulu-Natal, Durban, South Africa; 3 Division of Infection and Immunity, University College London, London, UK; 4 London School of Hygiene and Tropical Medicine, London, UK; 5 Department of Global Health, University of Washington, Seattle, USA; 6 Division of Infectious Diseases, Massachusetts General Hospital, Boston, MA USA; 7 Harvard Medical School, Boston, MA USA; 8 Department of Medicine, Division of Infectious Diseases, University of Washington, Seattle, USA

**Keywords:** HIV, ART initiation, Disclosure, South Africa

## Abstract

Accurate reporting of antiretroviral therapy (ART) uptake is crucial for measuring the success of epidemic control. Programs without linked electronic medical records are susceptible to duplicating ART initiation events. We assessed the prevalence of undisclosed ART use at the time of treatment initiation and explored its correlates among people presenting to public ambulatory clinics in South Africa. Data were analyzed from the community-based delivery of ART (DO ART) clinical trial, which recruited people living with HIV who presented for ART initiation at two clinics in rural South Africa. We collected data on socioeconomic factors, clinical factors, and collected blood as part of study screening procedures. We estimated the proportion of individuals presenting for ART initiation with viral load suppression (< 20 copies/mL) and fitted regression models to identify social and clinical correlates of non-disclosure of ART use. We also explored clinical and national databases to identify records of ART use. Finally, to confirm surreptitious ART use, we measured tenofovir (TDF) and emtricitabine (FTC) levels in dried blood spots. A total of 193 people were screened at the two clinics. Approximately 60% (n = 114) were female, 40% (n = 78) reported a prior HIV test, 23% (n = 44) had disclosed to a partner, and 31% (n = 61) had a partner with HIV. We found that 32% (n = 62) of individuals presenting for ART initiation or re-initiation had an undetectable viral load. In multivariable regression models, female sex (AOR 2.16, 95% CI 1.08–4.30), having a prior HIV test and having disclosed their HIV status (AOR 2.48, 95% CI 1.13–5.46), and having a partner with HIV (AOR 1.94, 95% CI 0.95–3.96) were associated with having an undetectable viral load. In records we reviewed, we found evidence of ART use from either clinical or laboratory databases in 68% (42/62) and detected either TDF or FTC in 60% (37/62) of individuals with an undetectable viral load. Undisclosed ART use was present in approximately one in three individuals presenting for ART initiation or re-initiation at ambulatory HIV clinics in South Africa. These results have important implications for ART resource use and planning in the region. A better understanding of reasons for non-disclosure of ART at primary health care clinics in such settings is needed.

## Background

South Africa has the highest number of people living with HIV (PLWH) in the world with an estimated 7.1 million PLWH [1]. Of the 7.1 million PLWH in South Africa, approximately 56% are on antiretroviral therapy (ART), making South Africa home to one of the largest ART programs in the world [1]. The ART program has been largely financed from its own domestic resources. In 2017 the country invested more than $1.54 billion annually to run its HIV programmes [2]. A critical element of the long-term response to the HIV epidemic in South Africa will be the sustainability of this program, and ability to accurately diagnose, treat, and sustain virologic suppression among those infected.

According to the Joint United Nations Programme on HIV/AIDS (UNAIDS) 2020, South Africa’s 90-90-90 progress stands at 92-70-64 [3]. Reliable estimates of HIV diagnoses and ART use among PLWH are needed to monitor progress towards fast-track targets set by UNAIDS to control the HIV pandemic by 2030 [4]. Similarly, accurate measurement of population-level ART use is needed to evaluate the programmatic efforts [5]. Methods used to measure these estimates include population-based demographic health surveys and health management information systems data from clinics [6]. The validity of this data is crucial to the accurate estimation of HIV program success and gaps. Of late, a handful of studies have demonstrated appreciable rates of virologic suppression and non-disclosure of ART use among individuals putatively presenting to care for treatment [7–13]. According to Fogel et al. undisclosed ART use is the use of “off study” antiretrovirals (ARVs) before enrolment into a clinical trial which may confound study outcomes [8]. This could have major implications if this practise is also found in people initiating ART in public health settings. Under-reporting of ART use in the community and clinics runs the risk of both mis-estimating true rates of treatment access, while duplicating ART initiation events for those who present repeatedly as new patient encounters. However, less is known about undisclosed ART use from public health clinics (PHCs) as most studies have been reported from a community and randomised clinical trial setting. Manne-Goehler et al. reported that ART denial could result in underestimates of ART coverage during efforts to monitor and evaluate progress toward international treatment targets [13]. That study investigated ART denial from home-based testing, however relatively little is known about this phenomenon in primary health care clinics, in which people are presenting for ART care initiation.

We recently completed a clinical trial of community delivery of ART in rural South Africa. We recruited treatment naïve or defaulted individuals at HIV clinics for enrolment in the study. We used this enrolment visit to assess virologic suppression rates among individuals presenting for new or resumption of HIV care, and to identify correlates of non-disclosure of active ART use in this population. We hypothesized that undisclosed ART use would be common amongst those who had indicated to have previously tested positive but had defaulted treatment and would be uncommon amongst those who indicated to be first time HIV testers.

## Methods

### Study Design

**Fig. 1:**
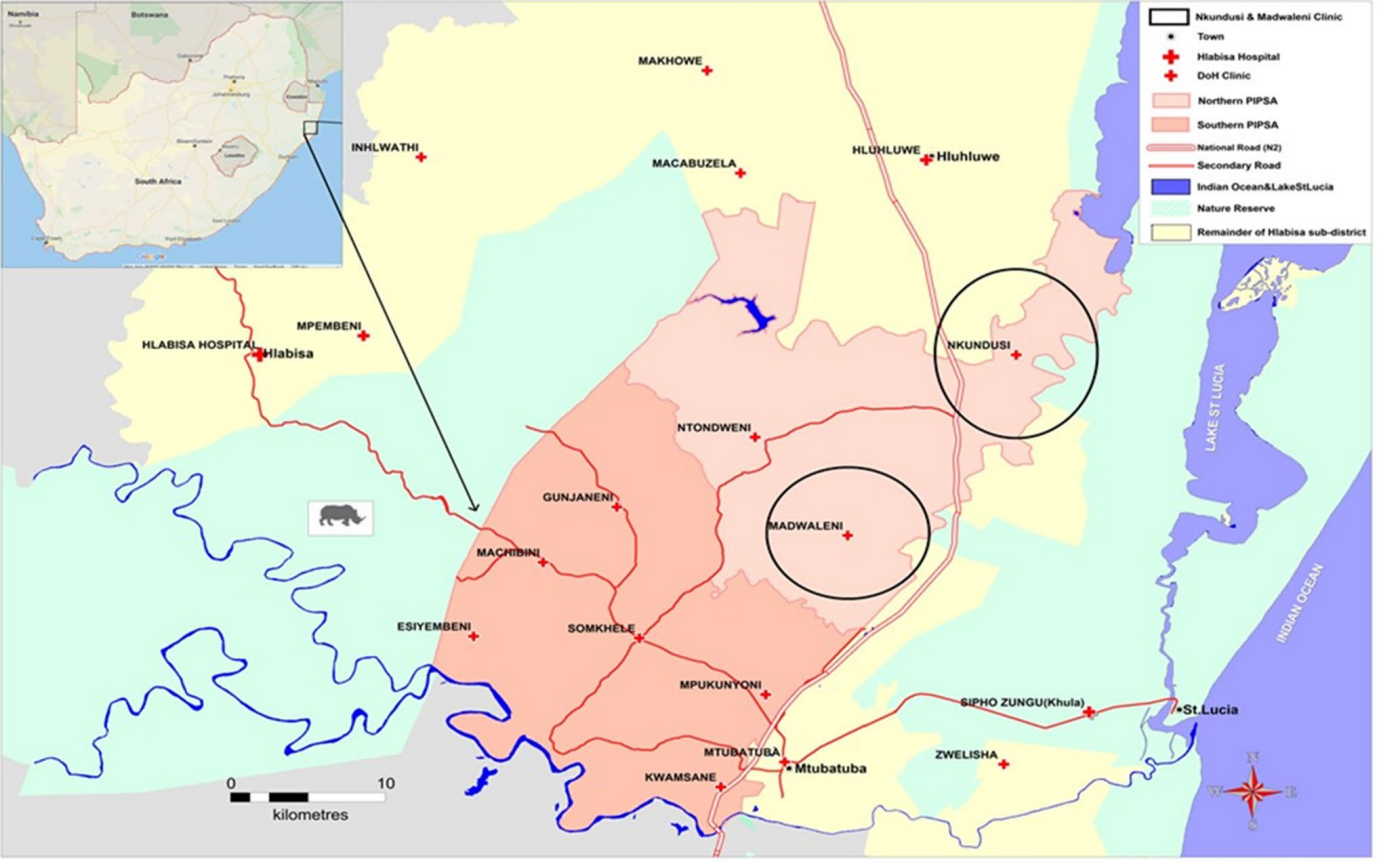
Hlabisa sub-district, uMkhanyakude district, Northern KwaZulu-Natal, South Africa

This was a cross-sectional analysis of data collected as part of the delivery optimization of antiretroviral therapy (DO-ART) clinical trial in 2 PHC clinics in uMkhanyakude district: Nkundusi and Madwaleni clinics (Fig. 1). Full details of that study have been published previously [14]. DO-ART recruited PLWH who self-reported as ART naïve or having defaulted for a minimum of three months from two clinical and community sites in South Africa. Individuals who tested HIV-positive at clinics and not currently in care were screened for study eligibility. At the study screening visit, potential participants were assessed for the following eligibility criteria: being at least 18 years old, notably no prior history of ART use or no active use in the past 90 days, CD4 cell count > 100 cells/μL, not being pregnant, and no evidence of active tuberculosis. This analysis includes all individuals who were referred for study screening after a positive HIV test at one of the two clinics.

### Study Procedures

#### Data and Specimen Collection

Screened participants completed a questionnaire to collect data on sex, age, clinic site, prior HIV testing, disclosure of their HIV serostatus to others, and whether they had a partner living with HIV. Blood was collected for dried blood spot (DBS) preparation, which was used to confirm HIV-1 RNA viral load for study eligibility. The threshold used to determine viral suppression was > 20 copies/mL. Blood was also collected for point of care Pima CD4 [15] count testing and Stat Sensor creatinine [16] and urine b-HCG pregnancy tests [17] for women were also collected.

We also reviewed individual records for each participant using two national databases and one research database to assess for prior ART use. A previous ART history was determined if there was any prior evidence of ART use in the National Health Laboratory Service (NHLS) database, the TIER.net clinical database, or the Africa Health Research Institute (AHRI) clinical information systems database [18–20]. We used the participant’s name, sex and date of birth as identifiers during our search.

Finally, to validate undisclosed ART use among individuals with an undetectable viral load, we tested for the presence of tenofovir (TDF) and emtricitabine (FTC) in DBS at Neuberg Global Laboratories, Durban South Africa. Drug levels were determined with the use of high performance liquid chromatography (HPLC) coupled to mass spectrometry [21, 22].

### Statistical Analysis

Our outcome of interest was ART non-disclosure, which we defined in all individuals who presented for initiation or re-initiation of HIV care with an undetectable viral load. We estimated the proportion of people presenting to care with an undetectable viral load based on their screening viral load assay. We then summarized sociodemographic and clinical characteristics for the total cohort then divided the cohort into individuals who did and did not have a detectable viral load. Next, we fitted logistic regression models, with virologic suppression as the outcome of interest, and the following variables as exploratory variables of interest: sex, age, distance from the clinic (distance between the clinic was categorized as the straight-line distance, as calculated using the difference in global positioning system (GPS) waypoints, between the center point of the village where the participant lives and the HIV clinic where they were accessing care), clinic site was dichotomized as Nkundusi versus Madwaleni clinic, conduct of prior HIV test dichotomized as ever versus never previously tested for HIV, receipt of a prior HIV positive test dichotomized as a receipt versus not of a prior positive HIV test, among those who had been previously tested for HIV, participants were considered to have disclosure of their HIV status if they reported disclosing it to at least one other person. All others were considered to be non-disclosed, and reporting of a partner living with HIV. We then fitted a multivariable regression model with any variables that reached a statistical significant of *P* < 0.25 in the univariable model. To calculate the distance participants traveled to the clinics, we used data from the AHRI Demographic Health and Surveillance Survey [23] which includes geographical information system (GIS) data on all households and clinics in the region. Finally, we compared the proportion who were undetectable by this assay who (1) had evidence of active ART use by DBS pharmacologic testing, measured by TDF and FTC assays and (2) through a record from electronic data systems including TIER.net, NHLS, or the AHRI clinical database. Statistical analysis was performed using Stata Version 16.0.

## Results

**Fig. 2:**
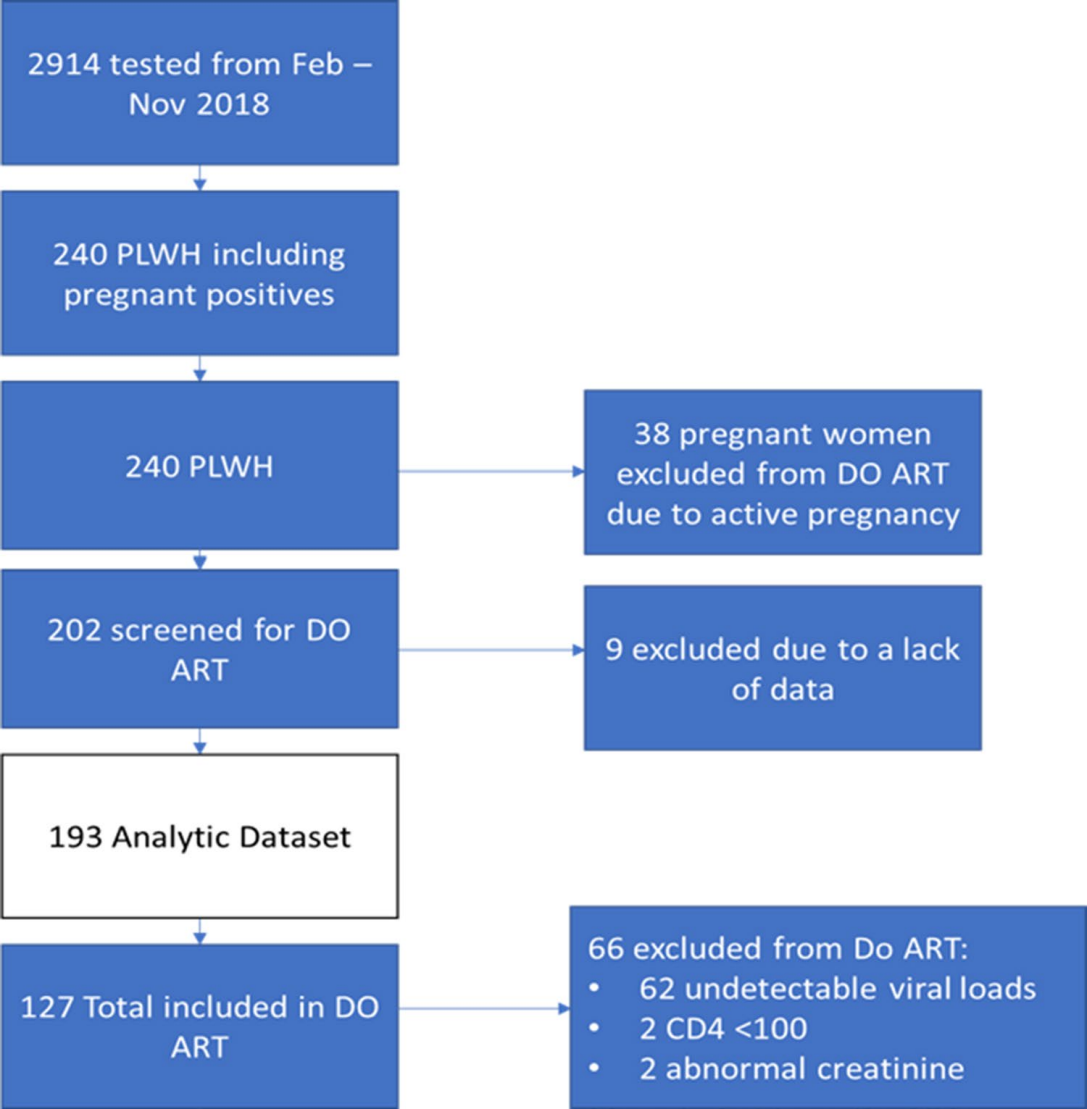
Flow chart of those who tested HIV from February–November 2018 at the two DO-ART study clinics

A total of 202 people tested positive and were screened for DO-ART at the two enrollment clinics (Fig. 2). Nine were excluded from the sample because no baseline data was collected, leaving a total of 193 individuals in this sub-study (Table 1). Approximarely 60% (n = 114) were female, 40% (n = 78) reported a prior HIV test, 23% (n = 44) had disclosed to a partner, and 31% (n = 61) had a partner with HIV.

**Table 1:** Participant characteristics

Characteristics	Presented with virologic suppression (n = 62)	Presented with a detectable viral load (n = 131)	*P* value
Female sex (n, %)	42 (21.7%)	72 (37.3%)	0.092
Age (n, %)			
18–29	31 (16.0%)	57 (29.5%)	0.607
30–49	28 (14.5%)	64 (33.1%)	0.607
> 50	3 (1.5%)	10 (5.1%)	0.607
Distance to clinic (n, %)			
< 3 km	13 (6.7%)	17 (8.8%)	0.344
3–5 km	39 (20.2%)	93 (48.1%)	0.344
≥ 5 km	10 (5.1%)	21 (10.8%)	0.344
Prior HIV test (n, %)			
Don’t know	1 (0.5%)	0 (0.0%)	0.041
No	29 (15.0%)	84 (43.5%)	0.041
Yes	31 (16.0%)	47 (24.3%)	0.041
Aware of HIV status (n, %)			
No, found out today	34 (17.6%)	93 (48.1%)	0.159
Yes, I knew my status	21 (10.8%)	28 (14.5%)	0.159
Yes, wanted to confirm	6 (3.1%)	9 (4.6%)	0.159
Yes, didn’t disclose	0 (0.0%)	1 (0.5%)	0.159
Last HIV result disclosed (n, %)			
Decline to answer	1 (0.5%)	3 (1.5%)	0.379
Don’t know	1 (0.5%)	1 (0.5%)	0.379
Yes	21 (10.8%)	23 (11.9%)	0.379
No	8 (4.1%)	20 (10.3%)	0.379
Partner living with HIV (n, %)			
Declined to answer	3 (1.5%)	6 (3.1%)	0.037
Don’t know	24 (12.4%)	79 (40.9%)	0.037
I think so	2 (1.0%)	5 (2.5%)	0.037
Don’t think so	2 (1.0%)	0 (0.0%)	0.037
No	6 (3.1%)	11 (5.6%)	0.037
Yes	24 (12.4%)	30 (15.5%)	0.037

**Fig. 3:**
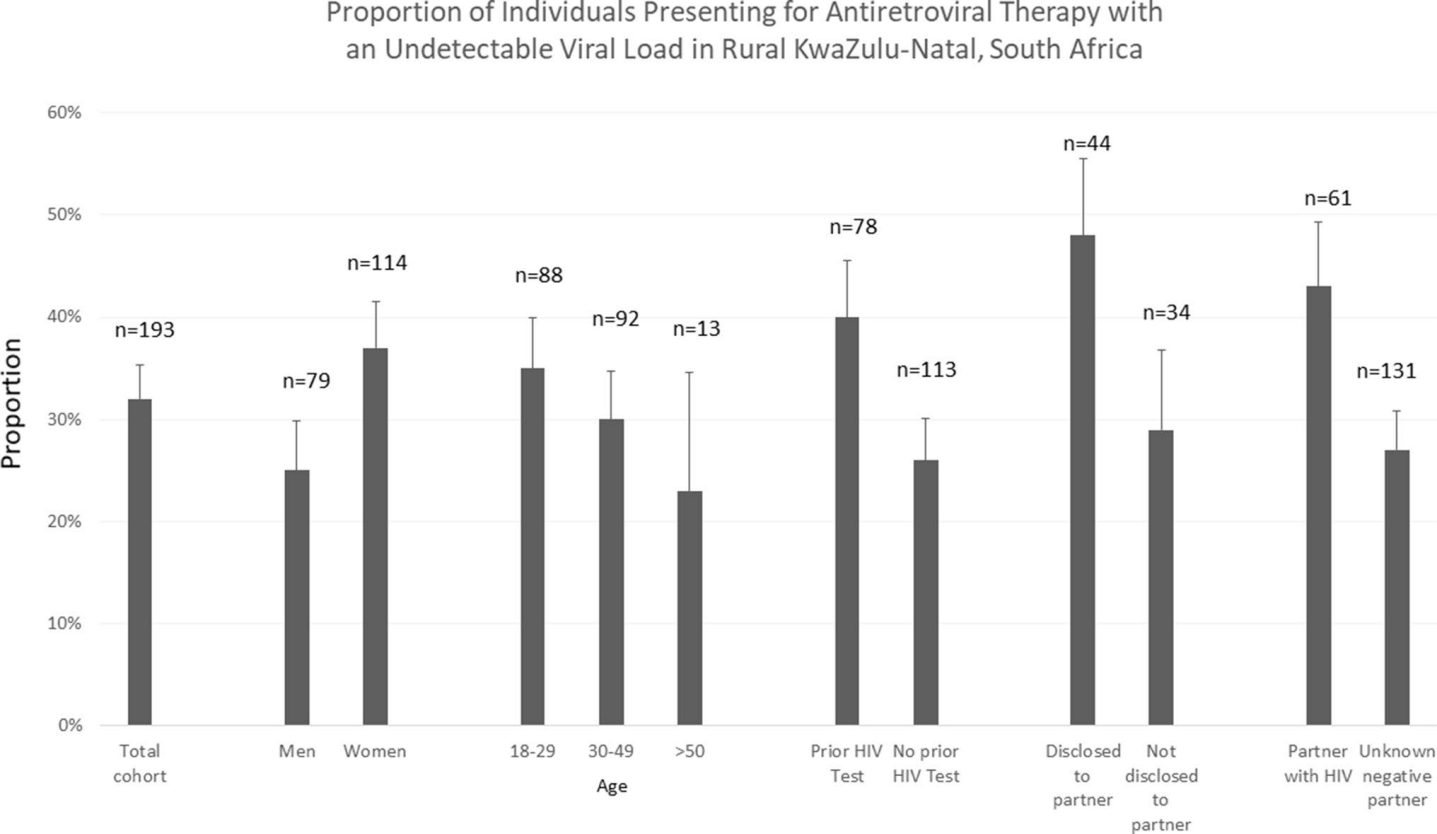
Proportion of individuals presenting for antiretroviral therapy with an undetectable viral load in rural KwaZulu-Natal, South Africa

In the total sample, 32% (n = 62) of individuals presenting for ART initiation or re-initiation had an undetectable viral load (Fig. 3). In multivariable regression models, female sex (AOR 2.16, 95% CI 1.08–4.30), having a prior HIV test, having disclosed their HIV status (AOR 2.48, 95% CI 1.13–5.46), and having a partner with HIV (AOR 1.94, 95% CI 0.95–3.96) were associated with having an undetectable viral load (Table 2).

**Table 2:** Univariable and multivariable logistics regression models with virological suppression at the time of clinic presentation as outcome of interest

Characteristic	Univariable models		Multivariable models	
	OR (95% CI)	*P* value	AOR (95% CI)	*P* value
Female	1.72 (0.91–3.24)	0.09	2.16 (1.08–4.30)	0.030
Age				
18–29	REF			
30–49	0.80 (0.43–1.50)	0.49		
≥ 50	0.55 (0.14–2.15)	0.39		
Distance from clinic				
< 3 km	REF		REF	
3–5 km	0.54 (0.24–1.23)	0.14	0.49 (0.20–1.18)	0.120
≥ 5 km	0.69 (0.22–2.09)	0.51	0.70 (0.23–2.13)	0.540
Clinic				
Madwaleni	REF			
Nkundusi	1.26 (0.66–2.41)	0.47		
HIV testing and disclosure status				
No prior positive HIV test	REF		REF	
Prior HIV test, non-disclosed^a^	1.20 (0.51–2.82)	0.67	1.35 (0.56–3.28)	0.500
Prior HIV test, disclosed	2.64 (1.27–5.27)	0.01	2.48 (1.13–5.46)	0.020
Partner living with HIV	2.03 (1.07–3.85)	0.03	1.94 (0.95–3.96)	0.070

^a^This category includes individuals who reported they did not know if they had disclosed their HIV status or who declined to answer the disclosure question

**Fig. 4:**
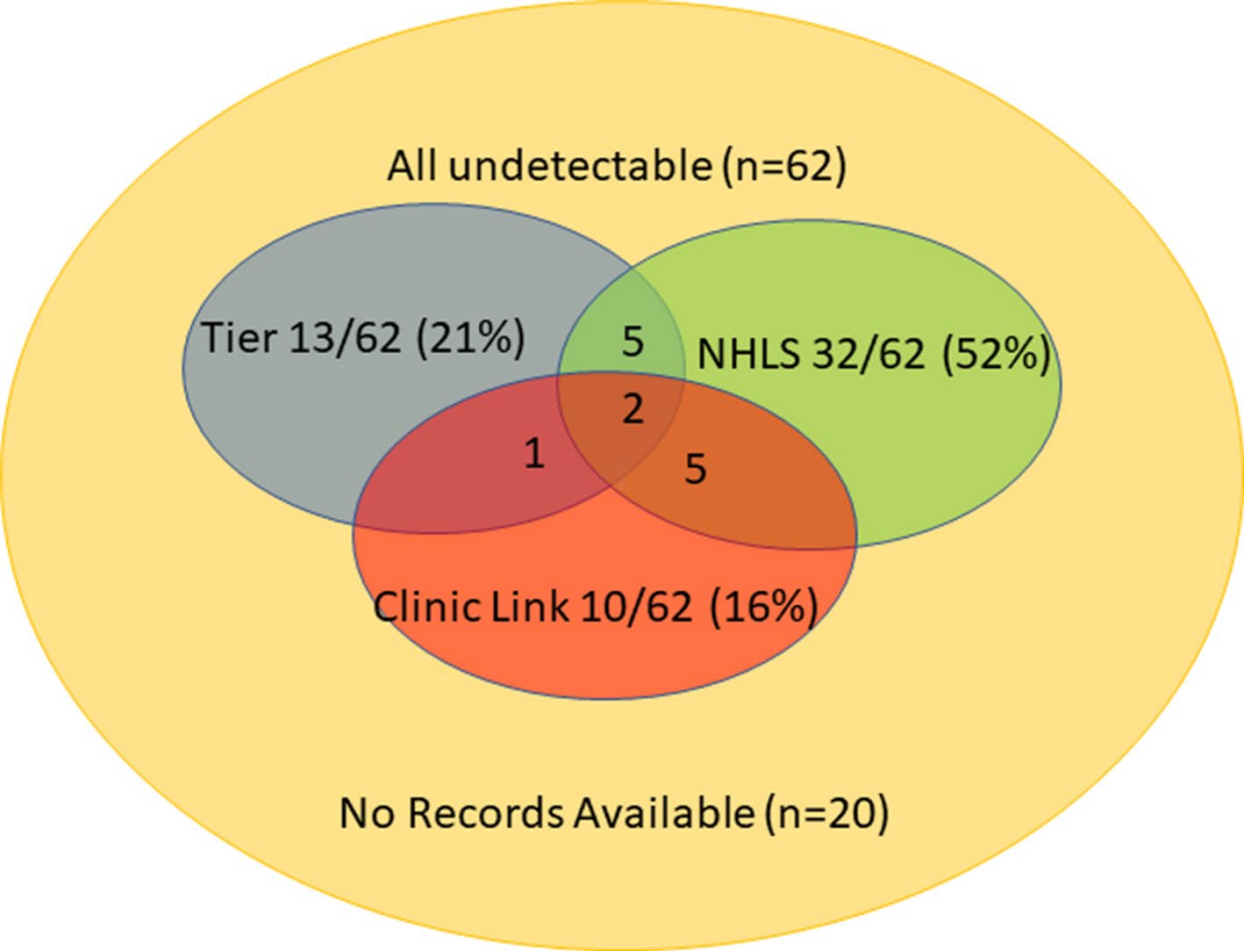
Availability of electronic medical records demonstrating prior ART use in individuals with an undetectable viral load at the time of presentation to care

When we explored medical record systems to investigate if any had prior evidence of ART use, we found that 68% (42/62) had evidence of prior ART use in at least one database (Fig. 4). The National Health Laboratory Services (NHLS) database was found to be the most sensitive of the systems identifying previous ART history in 52% (32/62) of individuals, followed by TIER.net at 21% (13/62), and the AHRI Clinic link database at 16% (10/62).

Finally, using pharmacologic drug level testing to explore recent ART use in those with an undetectable viral load, we found that 60% (37/62) were found to have detectable levels of either TDF or FTC in DBS specimens. Based on drug half-lives of these two drugs, this suggests that at least 60% of the sample presenting for ART initiation had taken ART in the past 10–17 h.

## Discussion

In this study in rural KwaZulu-Natal among individuals with HIV infection presenting for ART initiation, we found that undisclosed ART use with resulting undetectable viral loads were present in approximately one in three individuals. These results have implications for both epidemic monitoring and resource allocation for HIV programs in the region. First, commonly used indicators to report new ART initiations could be significantly misestimated if our results are reflective of a widespread phenomenon. Second, the over-prescription of ART could reflect a misuse of scarce resources. Finally, if these individuals are sharing their medication with family members or others, this could potentially have implications related to the treatment of undocumented HIV outside of clinical settings in the region.

Other studies have also demonstrated that non-disclosure of ART use can be common in community settings and in clinical trials [7–13]. For example, Fogel reported that off-study ART use was documented in approximately half of participants with viral suppression in the HIV prevention trials network (HPTN) 052 study [8]. Manne-Goehler found that one in three individuals with detectable ART in their blood denied current ART use during a community-based population survey in rural Mpumalanga, South Africa [13]. Huerga reported that up to 70% of individuals did not reveal they were taking ART at enrollment in a study which used a comparison of self-report and ARV detection to inform estimates of ARV therapy coverage, viral load suppression and HIV incidence in Kwazulu-Natal, South Africa [7]. A key difference between these prior studies and ours is the clinical facility setting of our study. We found a surprisingly high prevalence of undetectable viral loads and evidence of active ART use among individuals reporting to a public clinic and requesting to initiate ART. No studies to our knowledge have investigated this phenomenon in a public health care setting previously, where denial of ART use can have implications related to programmatic quantification of ART use and duplication of prescriptions of ART.

A number of factors were associated with non-disclosure of ART use in our study. We found that women, those with disclosure of a prior HIV test and those with a partner with HIV all correlated with having an undetectable viral load. Nearly 40% of women and 50% of those who had disclosed previously had an undetectable viral load, which might signal a strategy to consider reviewing medical records or viral load testing in certain sub-groups prior to ART initiation. We hypothesize that the presence of a partner with HIV might correlate with virologic suppression at presentation in individuals registering for care to provide ART for family members. The fact that disclosure of a prior HIV test also correlated with having undetectable viral loads strengthens our hypothesis as it has been reported that disclosure to a partner reduces the risk of an elevated viral load [24]. The exercise of sharing medication is something which has been previously reported [25] and our assumption is that the practise of undisclosed ART use, which was found to be prevalent in our research setting, is due to individuals sharing their treatment. Does having extensive social networks with PLWH promote undisclosed ART use? Future research should explore this possible phenomenon.

To validate our findings, we explored other sources of evidence in support of ART use, and found that most people with a detectable viral load had evidence of ART use in medical databases (68%) and during pharmacologic testing for ARV drugs (60%). Notably, the public databases that are used in KwaZulu-Natal for registration of ART use are largely inaccessible in real time by PHC nurses, due to lack of internet based access (for TIER.net), lack of user rights (for NHLS), or lack of access to computers or the internet at clinics. In contrast, these systems are generally available in the Western Cape [26]. Indeed, the fact that we found evidence of ART use in 68% of the databases suggests that if South Africa had a national health identifier, internet-based data systems, and computer and internet access in clinics accessible to nurses, that duplicative ART registrations use could potentially be prevented in many cases. We suspect that ARVs were found in less than 100% of those with virologic suppression because we tested only for TDF and FTC, which have half-lives ranging from 10 to 17 h [27]. Efavirenz (EFV), which has a longer half-life of between 40 and 55 h was not analysed. Participants who had not taken their treatment in the 24 h prior to presentation might not have been detected through our testing. Another possible explanation for why ARVs were not detected in all is that the participants could have been on regimens that did not include TDF and FTC.

This study was limited to recruitment from two clinics in rural South Africa and might not be generalizable to other areas of South Africa or elsewhere. We also note that survey responses on disclosure, prior HIV testing, and partners with HIV were taken by self-report and might be susceptible to social desirability bias. In this quantitative study, we were unable to determine the reasons for non-disclosure of ART history by participants to health care providers. Importantly, at the time of presentation to the clinic for HIV testing and ART initiation, the participants were not aware of the DO-ART study. Thus, motivation to join a research study are not likely to have influenced our findings. The potential reasons of having a high prevalence of undisclosed ART use in our setting could be attributed to HIV literacy, where individuals could have been unaware that they were taking ART, or it could be attributed to problems within the health system which makes PLWH not want to reveal their ART histories. To better understand the reasons behind non-disclosure we identified here, future qualitative work will be needed.

In summary, we found that 32% of individuals presenting for initiation or re-initiation at HIV ambulatory clinics in South Africa had undetectable viral loads. The fact that we found evidence of prior ART use in 68% regional clinical databases and evidence of TDF and/or FTC in 60% of the samples validates the fact that undetectable viral loads in these cases were due to undisclosed ART use among known HIV-positive individuals. These results require attention both by the research community to better explore the causes of non-disclosure of ART use, and by policy makers as they have important implications for ART reporting, resource use and planning in the region.
